# Constitutive Model and Fracture Failure of Sandstone Damage under High Temperature–Cyclic Stress

**DOI:** 10.3390/ma15144903

**Published:** 2022-07-14

**Authors:** Ji’an Luo, Jun He

**Affiliations:** 1 School of Mechanics and Photoelectric Physics, Anhui University of Science and Technology, Huainan 232001, China; 2 School of Civil Engineering, Anhui University of Science and Technology, Huainan 232001, China; hj14021522762022@163.com

**Keywords:** graded loading and unloading, damage constitutive model, Mohr–Coulomb strength criterion, thermal damage, damage constitutive evolution law

## Abstract

Deformation and damage characteristics of high-temperature rocks during underground coal gasification are the fundamental mechanical problems encountered in coal-bed gasification production. In order to study the characteristics of deformation and damage processes of rocks under the joint action of high temperature and high stress, a damage intrinsic model under the joint action of high temperature–cyclic loading is needed. In this paper, we used the damage mechanics theory to establish the damage instantiation model of rocks under the joint action of high temperature loads based on the Mohr–Coulomb strength criterion. It was found that the higher the temperature, the lower the strength of sandstone, the lower the peak stress, and the higher the peak strain, the peak stress decreased from 95.6 MPa at room temperature to 74.8 MPa at 400 °C to 49.5 MPa at 800 °C, and the peak strain increased from room temperature to 400 °C to 800 °C by 27.9% and 33.4%, respectively. With the increase in temperature, the internal microcracks of sandstone increased and expanded, which caused the degree of damage to intensify, and the macroscopic expression was the reduction in strength and stiffness of the sandstone. The rock went through four stages from loading to damage, including damage weakening, plastic deformation, strain softening, and residual deformation. The four types of fractures that led to the overall rupture of the rock were open fracture, secondary coplanar fracture, secondary inclined fracture, and oblique fracture. The damage intrinsic model constructed in this paper could better reflect the damage process of thermally damaged sandstone under the action of periodic loading, and had certain rationality. The damage intrinsic evolution curve, as well as the damage mechanism of sandstone under the action of macroscopic high temperature-loading, were discussed from the perspective of microscopic damage evolution, and the fracture extension pattern and penetration mechanism of the rock under different temperatures were analyzed. The research results provide an important reference for the design and engineering application of gasifiers in coal-bed underground gasification projects.

## 1. Introduction

Underground coal gasification is an effective new green, low-carbon, and environmentally friendly means of mining deep coal resources. The formation and diffusion of the combustion void area in the process of underground coal gasification is a dynamic process that is the result of the continuous expansion of the gasification channel with combustion and oxidation, accompanied by the generation of a large number of fractures and continuous expansion and collapse. If the fall space is too large, it may cause excessive destruction of the rock layer above the gasification area and the continuous development of fractures around the gasification area [1]. The rocks around the gasification zone have been subjected to high-temperature effects and are in a certain stress field that will inevitably be subjected to cyclic loading and unloading. Studying the damage constitutive model and damage constitutive evolution law of thermally damaged sandstone under the action of graded unloading can reflect the characteristics of the rock deformation and damage process, which is of certain theoretical and practical significance for the study of underground coal gasification.

Some researchers previously studied the mechanical properties and internal structure changes of rock under different temperatures [2,3,4,5,6,7]. Li Ximon et al. [8] analyzed the fatigue deformation and characteristics of rock under cyclic loading. Wen Tao et al. [9] proposed a damage evolution equation that can reflect the initial damage of rock. Some scholars proposed the concept of thermal damage and established the damage intrinsic model of rocks under the action of thermomechanical coupling [10,11,12]. Cao et al. [13,14] established a statistical constitutive model of damage softening that reflects the entire process of rock failure. Pan Jiliang et al. [15] proposed a damage ontological model combining hydrochemical damage with coupled damage of microcracks and macroscopic single fissures. Peng Zhixiong et al. [16] proposed a new model for the statistical damage constitution of rocks in deep strata under the assumptions that the neutron strength of rocks follows a two-parameter Weibull distribution and the neutron damage follows a modified Mohr–Coulomb strength criterion. Mansour Sabri et al. [17] experimentally investigated the effect of grain size on the damage mechanism and fracture toughness of rock specimens. T.D. Rathnaweera et al. [18] investigated the effect of extreme temperatures (from 25 °C to 1000 °C) and two cooling methods (fast and slow) on the mechanical behavior of clay-rich Hawkesbury sandstone under uniaxial conditions. Martin P. J. Schöpfer et al. [19] used the discrete element method (DEM) to investigate the dependence of elasticity, strength, and friction angle on porosity and crack density. Wang, S. et al. [20] considered the effects of the triple-shear Drucker–Prager yield criterion, compaction coefficient K, damage variable correction factor δ, and thermal damage variable *D_T_* to establish the intrinsic behavior of rock damage evolution under coupled temperature and load effects.

In this study, we first examined the physical and mechanical properties of sandstone at different temperatures. Uniaxial compression tests were conducted and the results were analyzed. Second, in order to study the characteristics of the rock deformation and damage process under high temperature–cyclic stress coupling, while considering the combined effect of temperature and cyclic load, using the principle of damage mechanics, and based on the Mohr–Coulomb strength criterion, we established a rock damage constitutive model under the combined action of high temperature–cyclic load and obtained the theoretical expressions of the model parameters. The high temperature and cyclic loading and unloading tests of sandstone were carried out, and the rationality of the constructed constitutive model were verified by comparing the test and theoretical curves. Third, in order to analyze the damage evolution curve and damage mechanism of sandstone under the macroscopic high temperature–load coupling from the perspective of microscopic damage evolution, the damage constitutive evolution law was analyzed, and the relationship between the microdamage evolution characteristics of thermally damaged sandstone under the action of graded loading and unloading and its macroscopic mechanical effects were explored. Finally, we analyzed the fracture extension pattern and penetration mechanism of sandstone at different temperatures.

## 2. High-Temperature Treatment and Mechanical Properties Test of Sandstone Specimens

In the following, we firstly processed the specimens to obtain the standard specimens; then the specimens were processed with a high-temperature heating device to reach the preset temperature; finally, the specimens were tested to obtain the mechanical property parameters and the stress–strain curves at different temperatures after applying the high temperature. The specific process was as follows.

### 2.1. Sampling and Processing of Sandstone Specimens

**Figure 1:**
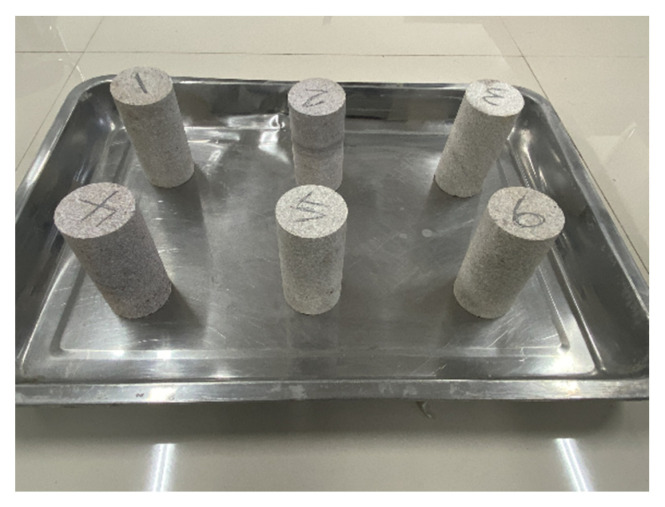
Sandstone specimens after high-temperature treatment.

The sandstone used in this study was taken from deep coal mine sandstone; rocks with good structural integrity and no appearance of damage were selected for sampling, and the test specimens were taken from the same rock block to enhance the comparability of the test results. According to the Rock Dynamic Properties Test Procedure, the rocks were cored, cut, ground, and polished perpendicular to the lamellar direction, with the nonparallel error at both ends of the sandstone specimens controlled within 0.05, and then processed into standard rock specimens of 50 mm in diameter and 100 mm in height to meet the test requirements. As shown in Figure 1.

### 2.2. High-Temperature Treatment

A chamber-type high-temperature sintering furnace heated the sandstone at high temperatures by means of thermocouples. The maximum heating temperature was designed to be 1200 °C, and the furnace was equipped with a temperature controller for setting the heating target temperatures of 400 °C and 800 °C.

For high-temperature heating of the sandstone specimens, the specimens were spaced evenly in the furnace chamber and the furnace door was closed, and then they were heated slowly at a heating rate of 10 °C/min. After heating to the preheating temperature, the sandstone was kept at a constant preheating temperature for 2 h to ensure more uniform heating inside and outside the sandstone specimens, and then the furnace door was slowly opened to allow the specimens to cool naturally to room temperature. Finally, the high-temperature-treated sandstone specimens were sealed and stored in sample bags, and relevant tests were carried out after all preparations were completed.

### 2.3. Mechanical Properties Test

**Figure 2:**
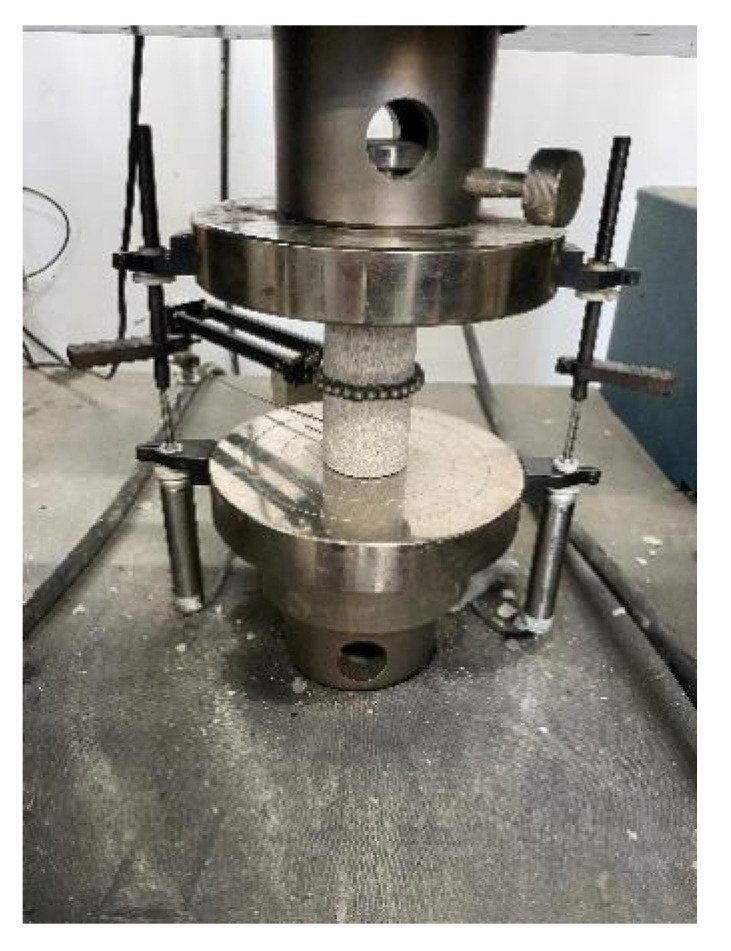
Graded loading and unloading tester.

Three sets of sandstone specimens treated at room temperature and high temperatures were subjected to conventional uniaxial compression tests and cyclic loading and unloading tests using a graded loading and unloading tester. Three sets of sandstone specimens treated at room temperature and high temperature were subjected to conventional uniaxial compression tests and cyclic loading and unloading tests using a graded loading and unloading tester. First, the unconfined compressive strengths of the sandstone specimens were measured using uniaxial compression tests at room temperature, 400 °C, and 800 °C to be approximately 90 MPa, 70 MPa, and 50 MPa, respectively, which provided the basis for the subsequent test parameters. The uniaxial compressive strengths of the sandstone at room temperature, 400 °C, and 800 °C were approximately 90 MPa, 70 MPa, and 50 MPa, respectively, so each set of specimens at different temperatures was tested at 7, 5, and 3 levels of addition and removal, respectively. The peak load stresses were about 30, 40, 50, 60, 70, 80, 90, and 100 MPa for sandstone at room temperature; the peak load stresses in sandstone at 400 °C are about 30, 40, 50, 60, 70 and 80 MPa; the peak load stresses in sandstone at 800 °C are about 30, 40, 50 and 60MPa. The specimens were damaged when the sandstone was loaded at room temperature, 400 °C and 800 °C to the seventh, fifth, and third levels, respectively. The test equipment is shown in Figure 2.

## 3. Analysis of Experimental Data

**Figure 3:**
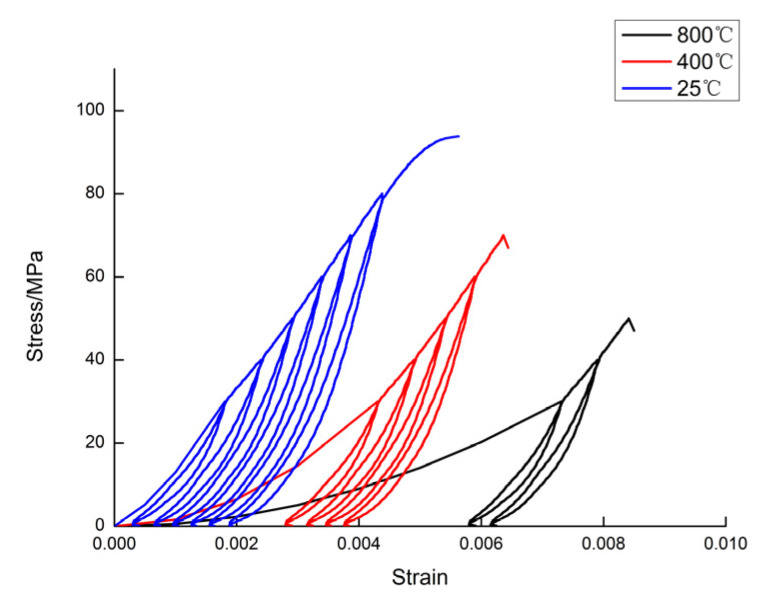
Sandstone stress–strain curve after different high temperatures.

In order to analyze the evolution law of axial stress and axial strain in the sandstone under the effect of graded unloading after application of high temperatures, Figure 3 shows the stress–strain curves of sandstone under the effect of graded loading and unloading after experiencing different high temperatures.

In the cyclic unloading curve section, it can be seen that although the stress and strain dropped to the minimum value, the curve could not return along the original path of the loading section, thus forming a hysteresis loop; the position of the hysteresis loop moved along the positive direction of strain, which was caused by the nonlinear characteristics of the rock itself. With each cycle of loading and unloading, new residual strains were generated, resulting in the accumulation of damage within the rock. As the temperature increased, the strength of the rock decreased, the peak stress decreased, the peak strain increased, and the stress–strain curve shifted toward the positive direction of the strain.

As the temperature increased, the number of additions and removals required for the rock to be damaged decreased, with 25 °C sandstone specimens experiencing damage at the seventh loading, 400 °C sandstone specimens at the fifth loading, and 800 °C sandstone specimens at the third loading.

The basic physical parameters of sandstone at different temperatures were obtained by uniaxial compression tests at different temperatures, as shown in Table 1.

In the table, we can see that the modulus of elasticity of the sandstone gradually decreased with the increase in temperature; Poisson’s ratio also showed an overall decreasing trend; the angle of internal friction increased with the increase in temperature between room temperature and 400 °C, and decreased with the increase in temperature between 400 °C and 800 °C. The cohesive force showed an overall increasing trend with the increase in temperature. Temperature caused the internal mineral particles of the sandstone to form microcracks due to the anisotropy and mismatch of thermal expansion and water loss, thus increasing the porosity, decreasing the elastic modulus, and decreasing the Poisson’s ratio, which deteriorated the mechanical properties of the sandstone. Higher temperatures lead to increases in the sliding resistance between adjacent soil layers, shear strength, internal friction angle, and cohesion.

## 4. Establishment of Damage Constitutive Model and Determination of Parameters

### 4.1. Establishment of Damage Constitutive Model

#### 4.1.1. Rock Damage Variables under Load

Using the principle of damage mechanics of continuous media, define *D* as the ratio of the number of microelements *n* that have been destroyed in the rock to the total number of microelements *N*; i.e.:

*D* = *n*/*N* (1)

According to the theory of statistical probability distribution, consider the rock damage probability as $P\left[f\left(\sigma^{*}\right)\right]=P\left[k_{0}\right]$. Thus, the general formula for the damage factor is:

(2) $$D=\int \begin{array}{c} k_{0}\\ 0 \end{array}P\left(x\right)dx$$

Assume that the damage probability density function of the microelement body of rock material is:

(3) $$P\left(F\right)=\frac{m}{F_{0}}{\left(\frac{F}{F_{0}}\right)}^{m-1}\exp\left[-{\left(\frac{F}{F_{0}}\right)}^{m}\right]$$

where: *F* is the distribution variable of microelement damage, and *m* and *F*_0_ are the Weibull distribution parameters.

So, the rock statistical damage variable under load can be expressed as:

(4) $$D=\int \begin{array}{c} F\\ 0 \end{array}P\left(x\right)dx=1-\exp\left[-{\left(\frac{F}{F_{0}}\right)}^{m}\right]$$

#### 4.1.2. Rock Damage Variables under the Effect of Temperature

Under high temperatures, the intensification of molecular thermal motion inside the rock and the inconsistency of thermal expansion of most minerals will lead to thermal stresses inside the rock, resulting in some microcracks; these cracks continue to expand, which in turn will lead to deterioration of the rock’s material properties. Therefore, to create a constitutive model of rock damage under load and temperature, it is necessary to consider temperature-dependent functions.

According to the principle of macroscopic damage mechanics, the damage to the rock by temperature can be characterized by macroscopic mechanical parameters, and the thermal damage variable [10] is defined as:

(5) $$D_{T}=1-\frac{E_{T}}{E_{0}}$$

where: *E_T_* is the modulus of elasticity of the rock at different temperatures, and *E*_0_ is the modulus of elasticity of the rock at room temperature.

#### 4.1.3. Total Rock Damage Variables Considering Load and Temperature

Since rocks exhibit different damage characteristics under load and temperature, the total damage of rocks under the combined action of load and temperature can be expressed according to the total damage variable derived from Huimei Zhang et al. [10], based on the strain equivalence principle as follows.

(6) $$D_{m}=D+D_{T}-DD_{T}$$

where: *D_m_* is the total damage variable of the rock under the joint action of load and temperature.

Substituting Equations (4) and (5) into Equation (6), the evolution equation of the total damage of the rock under the joint action of load and temperature is obtained as follows:

(7) $$D_{m}=1-\frac{E_{T}}{E_{0}}\exp\left[-{\left(\frac{F}{F_{0}}\right)}^{m}\right]$$

#### 4.1.4. The Constitutive Relationship of Rock Damage Considering Load and Temperature

*D* is the damage variable caused by the action of load, and *D_T_* is the damage variable caused by the action of high temperature. *D* can be obtained using the Weibull statistical probability function, while *D_T_* is not convenient to find. So, Huimei Zhang et al. [10] proposed that rocks experience temperature damage and load loss under temperature–load. The damage under the combined action of temperature and load and the nondamaged part share the axial stress. Therefore, it can be considered that the constitutive relationship of rock damage under the combined action of temperature and load is:

(8) $$\sigma_{1}=E_{T}\varepsilon_{1}\left(1-D_{m}\right)+GD_{m}+\mu \left(\sigma_{2}+\sigma_{3}\right)$$

where *G* is the temperature damage coefficient. When damage to the rock occurs, *D_m_* is 1, which can be found by bringing it into (8):

(9) $$G=\sigma_{r}-\mu \left(\sigma_{2}+\sigma_{3}\right)$$

where $\sigma_{r}$ is the residual stress of the rock. Based on the study by Wengui Cao et al. [13,14], which considered rock materials conforming to the Mohr–Coulomb criterion, the rock axial residual stress can be obtained as:

(10) $$\sigma_{r}=\frac{\left(1-\sin\varphi_{r}\right)\sigma_{3}+2C_{r}\cos\varphi_{r}}{1-\sin\varphi_{r}}$$

*C* is the cohesive force, thus:

(11) $$G=\frac{\left(1-\sin\varphi_{r}\right)\sigma_{3}+2C_{r}\cos\varphi_{r}}{1-\sin\varphi_{r}}-\mu \left(\sigma_{2}+\sigma_{3}\right)$$

Therefore, the rock damage principal structure relationship under the joint temperature-load action is:

(12) $$\sigma_{1}=\frac{E_{T}\left(E_{T}\varepsilon_{1}-G\right)}{E_{0}}\exp\left[-{\left(\frac{F}{F_{0}}\right)}^{m}\right]+G+2\mu \sigma_{3}$$

The Mohr–Coulomb strength criterion was obtained by predecessors after many previous experiments. This criterion conforms to the damage characteristics and mechanical properties of most rocks, so the microelement strength of rocks based on the Mohr–Coulomb strength criterion has good rationality and practicality.

The expression of the Mohr–Coulomb strength criterion is a function of:

(13) $$\sigma_{t}=\sigma_{1}-\frac{1+\sin\varphi }{1-\sin\varphi }\sigma_{3}$$

Thus, the rock microelement strength based on the Mohr–Coulomb strength criterion is:

(14) $$F=\sigma_{1}^{*}-\frac{1+\sin\varphi }{1-\sin\varphi }\sigma_{3}^{*}$$

where *ψ* is the angle of internal friction; $\sigma_{1},\sigma_{3}$ are the nominal maximum principal stress and minimum principal stress after correction for damage variables, respectively; and:

(15) $$\sigma_{i}^{*}=\frac{\sigma_{i}}{1-D}$$

So:

(16) $$\sigma_{1}=\frac{E_{T}\left(E_{T}\varepsilon_{1}-G\right)}{E_{0}}\exp\left\{-{\left[\left(\sigma_{1}^{*}-\frac{1+\sin\varphi }{1-\sin\varphi }\sigma_{3}^{*}\right)/F_{0}\right]}^{m}\right\}+G+2\mu \sigma_{3}$$

When the rock is in the uniaxial state, Equation (16) is transformed to obtain the rock intrinsic model under uniaxial action as:

(17) $$\sigma =\frac{E_{T}\left(E_{T}\varepsilon -G\right)}{E_{0}}\exp\left\{-{\left[E_{T}\varepsilon \left(1-\frac{1-3\sin\varphi }{1-\sin\varphi }\mu \right)/F_{0}\right]}^{m}\right\}+G$$

where

(18) $$G=\frac{2C_{r}\cos\varphi_{r}}{1-\sin\varphi_{r}}$$

### 4.2. Determination of Parameters

When the rock experiences damage, the stress–strain curve of the rock should satisfy the following conditions:

(19) $$\varepsilon_{1}=\varepsilon_{f},\sigma_{1}=\sigma_{f}$$

(20) $$\varepsilon_{1}=\varepsilon_{f},\frac{d\sigma_{1}}{d\varepsilon_{1}}=0$$

where $\varepsilon_{f},\sigma_{f}$ are the peak strain and peak stress, respectively.

Substituting $\varepsilon_{1}=\varepsilon_{f},\sigma_{1}=\sigma_{f}$ into Equation (12), we get:

(21) $$\exp\left(\frac{F_{f}}{F_{0}}\right)=\frac{E_{T}\left(E_{0}\varepsilon_{f}-G\right)}{E_{0}\left(\sigma_{f}-G-2\mu \sigma_{3}\right)}$$

where $F_{f}$ is the *F* value corresponding to the peak.

Substituting $\sigma_{1}^{*}=E_{t}\varepsilon_{1}+\mu \left(\sigma_{2}+\sigma_{3}\right)$ into Equation (14), we get:

(22) $$F=E_{T}\varepsilon_{1}+Y\sigma_{3}$$

where $Y=2\mu -\frac{1+\sin\varphi }{1-\sin\varphi }$, according to the assumption of Huimei Zhang et al. [10]; $\sigma_{i}$ is a function of $\varepsilon_{i}$ and *n*. Assuming that $F_{0}$, *m*, $E_{T}$, and *µ* are only a function of stress $\sigma_{3}$ and *n*, the functional relationship between $F_{0}$, *m* is obtained as:

(23) $${\left(\frac{F_{f}}{F_{0}}\right)}^{m}=\frac{E_{T}\varepsilon_{f}+Y\sigma_{3}}{m\left(E_{T}\varepsilon_{f}-G\right)}$$

By combining (21) and (23), the expressions of the model parameters are obtained as:

(24) $$m=\frac{E_{T}\varepsilon_{f}+Y\sigma_{3}}{\left(E_{T}\varepsilon_{f}-G\right)\ln\frac{E_{T}\left(E_{T}\varepsilon_{f}-G\right)}{E_{0}\left(\sigma_{f}-G-2\mu \sigma_{3}\right)}}$$

(25) $$F_{0}=F_{f}{\left[\frac{m\left(E_{T}\varepsilon_{f}-G\right)}{E_{T}\varepsilon_{f}+Y\sigma_{3}}\right]}^{\frac{1}{m}}$$

Using Equations (7), (22), (24) and (25), the total damage evolution equation can be obtained as:

(26) $$D_{m}=1-\frac{E_{T}}{E_{0}}\exp\left[-W\ln\frac{E_{T}\left(E_{T}\varepsilon_{f}-G\right)}{E_{0}\left(\sigma_{f}-G-2\mu \sigma_{3}\right)}\right]$$

where $W={\left(\frac{E_{T}\varepsilon_{1}+Y\sigma_{3}}{E_{T}\varepsilon_{f}+Y\sigma_{3}}\right)}^{m}$.

## 5. Verification of the Damage Constitutive Model

**Figure 4:**
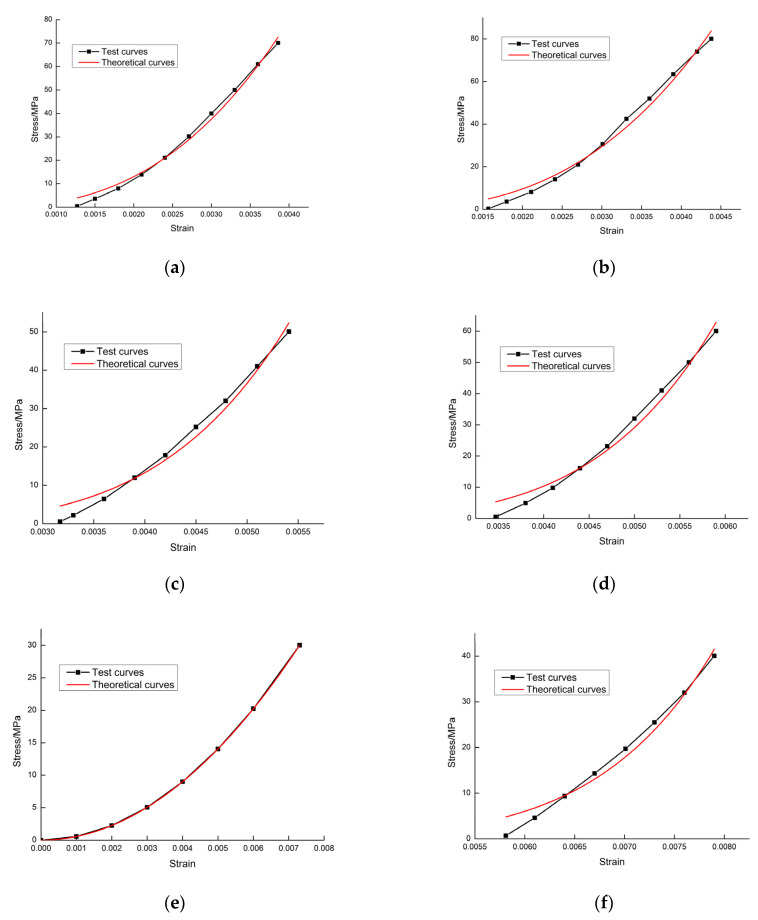
Comparison of theoretical and experimental curves during the loading phase: (**a**) 25 °C, 70 MPa level loading; (**b**) 25 °C, 80 MPa level loading; (**c**) 400 °C, 50 MPa level loading; (**d**) 400 °C, 60 MPa level loading; (**e**) 800 °C, 30 MPa level loading; (**f**) 800 °C, 40 MPa level loading.

**Figure 5:**
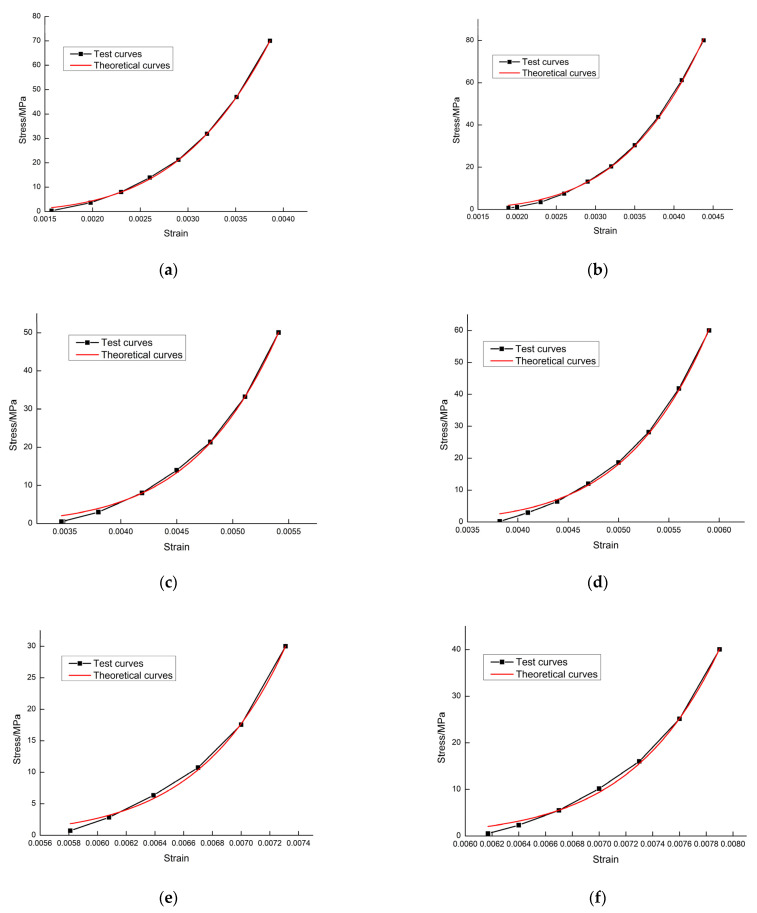
Comparison of theoretical and experimental curves during the unloading phase: (**a**) 25 °C, 70 MPa class unloading; (**b**) 25 °C, 80 MPa class unloading; (**c**) 400 °C, 50 MPa class unloading; (**d**) 400 °C, 60 MPa class unloading; (**e**) 800 °C, 30 MPa class unloading; (**f**) 800 °C, 40 MPa class unloading.

Using the experimental results shown in Figure 3, and then combining them with the calculation of Equation (17), several sets of theoretical curves of the rock damage constitutive model could be obtained for different cases at different temperatures. The six grade loading and unloading test curves for different temperatures and stress values shown in Figure 3 were selected and compared with the theoretical curve; each grade test curve corresponded to the loading curve and unloading curve. It can be observed in Figure 4 and Figure 5 that the theoretical curves of the rock damage intrinsic structure model established in this paper overlapped well with the experimental curves, thus verifying the rationality of this rock damage constitutive model.

## 6. Analysis of the Constitutive Evolution Law of Rock Damage

According to Equation (23) and the experimental data, we created the total damage evolution curve of sandstone under the action of high temperature–cyclic stress, and then selected the change curve of damage factor at the last loading stage of each temperature, and found that the total damage variables of the rock could be divided into four stages with the strain curve, including damage weakening stage, plastic deformation stage, strain softening stage, and residual deformation stage.

**Figure 6:**
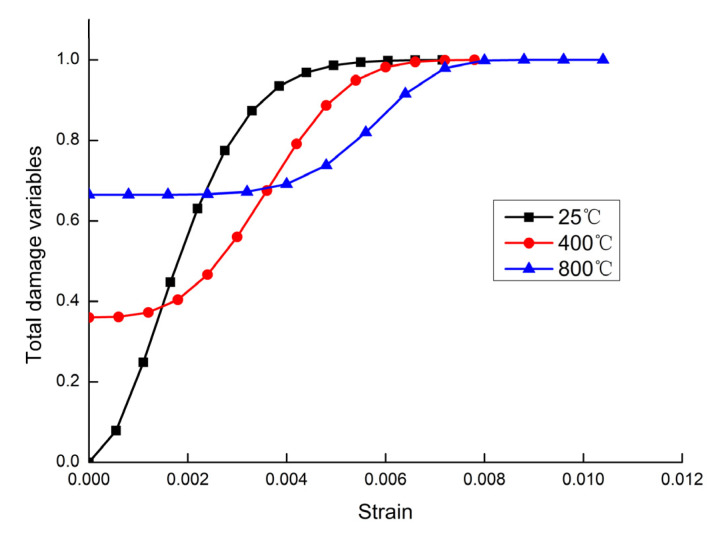
Total damage evolution curve of the sandstone at different temperatures.

The damage value corresponding to the strain of 0 in Figure 6 was the high-temperature damage value, and the high-temperature damage value increased with the increase in temperature. After experiencing the 400 °C and 800 °C temperatures, the high temperature damage variables of the sandstone were 0.36 and 0.665, respectively. Compared with room temperature, the increase in the high-temperature damage variable of the sandstone after experiencing the 800 °C temperature was 66.5%. When the damage variable tended to 1, the strength of the sandstone reached its peak, and damage occurred. The strain corresponding to the damage variable tending to 1 increased with the temperature, and the strain was about 0.0055 when the sandstone was damaged at room temperature, about 0.0061 when the sandstone was damaged at 400 °C, and about 0.0083 when the sandstone was damaged at 800 °C.

The total damage variables under the combined effect of load and temperature showed an overall increasing trend with the increase in the strain level. After applying the high temperature, the sandstone was weakened at the initial stage of loading, the microfractures inside the sandstone gradually closed, the density of the sandstone increased, and the strength increased. After that, the sandstone was in the plastic deformation stage: the fractures began to expand, and the damage began to evolve and expand until the damage accelerated. Then, the sandstone entered the strain softening stage, and the microfractures inside the sandstone continuously sprouted, expanded, and penetrated. Finally, the sandstone entered the residual deformation stage, and the total damage variable tended to 1. Macroscopic cracks occurred in the sandstone, and the sandstone’s strength reached its peak, causing damage.

With the increase in temperature, the compressively stable section of sandstone gradually increased, mainly due to the increase in temperature. The internal damage of sandstone intensified, increasing the probability of microfractures generated inside the rock, and therefore the more obvious the compressively stable section was after being compressed.

The damage variable corresponding to the strain value increased continuously with the increase in temperature, which indicated that the high temperature caused the intensification of molecular thermal movement inside the sandstone. The uncoordinated thermal expansion between a variety of mineral particles led to the thermal stress inside the sandstone, which generated microcracks and expanded continuously, causing the degree of damage to intensify, which was macroscopically manifested as a reduction in the strength and stiffness of the sandstone. After the sandstone entered the plastic deformation stage, the damage evolution curves were interwoven successively, and the damage variable decreased with the increase in temperature after interweaving, which was macroscopically manifested as the weakening of the sandstone’s resistance to deformation and more obvious plastic deformation.

The mechanical properties and damage expansion law of sandstone obtained from the damage intrinsic model of sandstone under high-temperature cyclic stress in this study were basically consistent with the experimental phenomena and analytical conclusions of other studies found in the literature, and the damage characteristics of the rock were basically consistent, indicating that the damage intrinsic model of sandstone under high-temperature cyclic stress established in this paper was reasonable.

## 7. Analysis of Rock Fracture Patterns

In this test, two groups of sandstone that experienced high-temperature heating at 400 °C and 800 °C, respectively, and sandstone at room temperature were subjected to cyclic addition and unloading tests. Through statistical analysis of sandstone damage patterns after the tests, it was found that there were four types of fractures that led to the overall rupture of the sandstone: (1) open fractures (Black Line); (2) secondary coplanar fractures (Red Line); (3) secondary inclined fractures (Blue Line); and (4) oblique fractures (Green Line).

**Figure 7:**
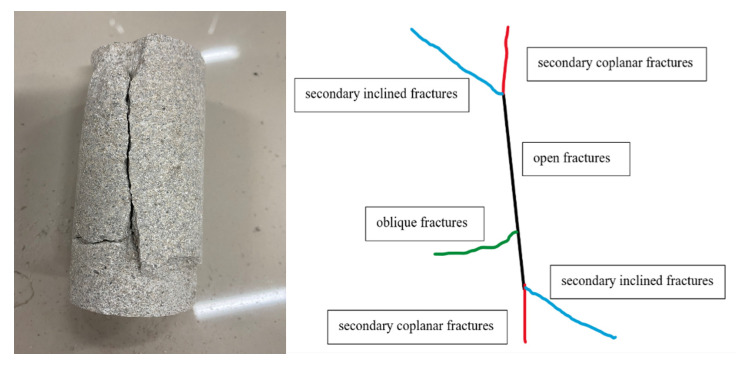
Fracture extension pattern of 25 °C sandstone specimen.

Under the normal temperature condition, the fracture extension of the sandstone specimens was composed of four fracture forms: open fracture, secondary coplanar fracture, secondary inclined fracture, and oblique fracture. Firstly, the open fissure was formed in the middle of sandstone, then the secondary coplanar fissure sprouted in the two tips of the open fissure, and the oblique fissure sprouted in the middle of the open fissure. With the increase in load, the secondary coplanar fissure was continuously expanded along the direction of the open fissure in the upper and lower tips of the open fissure, and the oblique fissure was expanded in the form of a zigzag line along the direction perpendicular to the open fissure, while the secondary coplanar fissure was expanded. At the same time of the expansion of the secondary coplanar fracture, a secondary inclined fracture appeared at the two tips and expanded along a certain angle direction with the open fracture, and finally the secondary coplanar fracture penetrated first and the sandstone specimen was destroyed. The upper and lower ends of the open fracture showed “Y” and inverted “Y” fracture expansion patterns, respectively. As shown in Figure 7.

**Figure 8:**
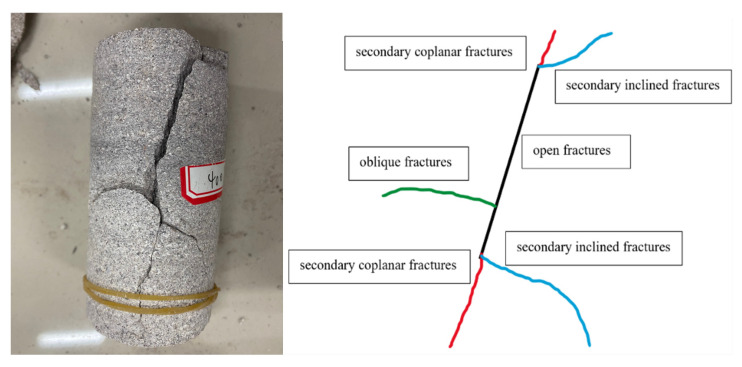
Fracture extension pattern of 400 °C sandstone specimen.

The fracture extension of the sandstone specimen after high-temperature heating treatment at 400 °C was composed of four fracture forms: open fracture, secondary coplanar fracture, secondary inclined fracture, and oblique fracture. Firstly, an open fissure was formed in the middle of sandstone, then the secondary coplanar fissure sprouted in the two tips of the open fissure, and the oblique fissure sprouted in the middle of the open fissure. With the increase in load, the secondary coplanar fissure kept expanding along the direction of the open fissure in the upper and lower tips of the open fissure, and the oblique fissure expanded along the direction perpendicular to the open fissure in the form of a zigzag line, while the secondary coplanar fissure expanded. At the same time of the expansion of secondary coplanar fracture, a secondary inclined fracture appeared at the two tips and expanded along a certain angle direction with the open fracture, and finally the secondary coplanar fracture penetrated first and the sandstone specimen was destroyed. The upper and lower ends of the open fracture showed “Y” and inverted “Y” fracture expansion patterns, respectively. As shown in Figure 8.

**Figure 9:**
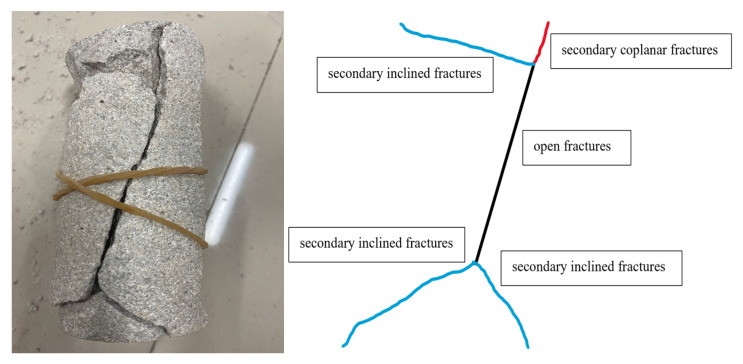
Fracture extension pattern of 800 °C sandstone specimen.

The fracture extension of the sandstone specimen after 800 °C high-temperature heating treatment was composed of three fracture forms: open fracture, secondary coplanar fracture, and secondary inclined fracture. Firstly, an open fissure was formed in the middle of the sandstone specimen, then secondary coplanar fissures sprouted in the upper tip of the open fissure. With the increase in load, secondary coplanar fissures kept expanding along the direction of the open fissure in the upper tip of the open fissure; while the secondary coplanar fissures expanded, secondary tilted fissures appeared in the upper tip of the open fissure, which expanded along the direction of a certain angle with the open fissure. Finally, the sandstone specimen was damaged, and the upper and lower ends of the open fissure showed “Y” and inverted “Y” fissure expansion patterns, respectively. As shown in Figure 9.

## 8. Conclusions

In this study, uniaxial compression tests and cyclic loading and unloading tests were performed on sandstone at three different temperatures. The results of the cyclic loading and unloading tests were in good agreement with the damage intrinsic model established in this paper, which predicted the process of rock deformation and damage and analyzed the damage evolution mechanism and fracture expansion pattern of the rocks at different temperatures. The following conclusions were drawn from the findings:

(1) By studying the physical and mechanical properties of sandstone at different temperatures, uniaxial compression tests and analysis revealed that due to the anisotropy and mismatch of thermal expansion and water loss, temperature led to the formation of microcracks; the porosity of mineral particles inside the sandstone increased; and at higher temperatures, the sliding resistance between adjacent soil layers increased and the shear strength increased, thus leading to different mechanical properties of the sandstone at different temperatures.
(2) In order to study the characteristics of the rock deformation and damage process under high temperature–cyclic stress coupling, we constructed a damage instantiation model for rocks under high temperature–cyclic stress coupling, which could express the stress–strain instantiation relationship of thermally damaged rocks under graded loading and unloading conditions more accurately. It was found that hysteresis loops occurred during the cyclic loading and unloading, and new residual strains were generated with each loading and unloading. The higher the temperature, the lower the strength of the sandstone, the lower the peak stress, and the higher the peak strain.
(3) By analyzing the damage evolution pattern of the sandstone, we found that with the increase in temperature, the pressure-dense stable section of the sandstone damage evolution curve gradually increased; the strain occurring when the sandstone reached damage increased with the increase in temperature; and from a macroscopic point of view, the strength and stiffness of the sandstone decreased with the increase in temperature. The damage intrinsic evolution curves revealed the corresponding fine mechanical and macroscopic damage behaviors of the sandstone, reflecting the characteristics of high-temperature damage and load damage on sandstone damage coupling and their influence on each other, and exposing the damage mechanism of sandstone under macroscopic high-temperature–load action from the fine damage evolution.
(4) When studying the crack-extension patterns and penetration mechanisms of rocks at different temperatures, four types of fractures leading to overall cracking in sandstone were found by analyzing the rock fracture patterns: open fractures, secondary coplanar fractures, secondary oblique fractures, and oblique fractures.

## Figures and Tables

**Table 1:** Basic physical parameters of sandstone at different temperatures.

Temperature/°C	Modulus of Elasticity/GPa	Poisson’s Ratio	Angle of Internal Friction/°	Cohesion/MPa
25 °C	37.948	0.261	45.28	15.555
400 °C	32.246	0.098	49.13	20.204
800 °C	12.696	0.105	46.32	26.485

## Data Availability

Not applicable.
